# Chitin Synthesis and Degradation in Crustaceans: A Genomic View and Application

**DOI:** 10.3390/md19030153

**Published:** 2021-03-15

**Authors:** Xiaojun Zhang, Jianbo Yuan, Fuhua Li, Jianhai Xiang

**Affiliations:** 1 CAS Key Laboratory of Experimental Marine Biology, Institute of Oceanology, Chinese Academy of Sciences, Qingdao 266071, China; xjzhang@qdio.ac.cn (X.Z.); yuanjb@qdio.ac.cn (J.Y.); fhli@qdio.ac.cn (F.L.); 2 Laboratory for Marine Biology and Biotechnology, Qingdao National Laboratory for Marine Science and Technology, Qingdao 266237, China; 3 Center for Ocean Mega-Science, Chinese Academy of Sciences, Qingdao 266071, China

**Keywords:** crustacean, chitin metabolism, chitin synthase, chitinase, chitin synthesis, degradation pathway

## Abstract

Chitin is among the most important components of the crustacean cuticular exoskeleton and intestinal peritrophic matrix. With the progress of genomics and sequencing technology, a large number of gene sequences related to chitin metabolism have been deposited in the GenBank database in recent years. Here, we summarized the genes and pathways associated with the biosynthesis and degradation of chitins in crustaceans based on genomic analyses. We found that chitin biosynthesis genes typically occur in single or two copies, whereas chitin degradation genes are all multiple copies. Moreover, the chitinase genes are significantly expanded in most crustacean genomes. The gene structure and expression pattern of these genes are similar to those of insects, albeit with some specific characteristics. Additionally, the potential applications of the chitin metabolism genes in molting regulation and immune defense, as well as industrial chitin degradation and production, are also summarized in this review.

## 1. Introduction

Chitin, a linear polymer of β-(1-4)-linked *N*-acetylglucosamines (GlcNAc), is the second most abundant natural polymer polysaccharide after cellulose and one of the largest unexploited renewable resources in existence [1]. This compound is mainly found in the shells of crustaceans, the exoskeletons of insects, the cuticle of other invertebrates, and the cell walls of fungi, providing the necessary rigidity and mechanical strength to support the structure and shape of living organisms [2,3].

Most studies on chitin have primarily focused on microorganisms and insects, however, the role of this important biopolymer in crustaceans has been largely overlooked. Crustaceans, such as shrimp and crabs, constitute the group with the largest chitin output, and their growth and development are majorly associated with the biosynthesis and modification of chitin. Therefore, understanding the biological pathways associated with chitin metabolism in crustaceans is crucial. Chitin metabolism is the result of the joint activity of the chitin synthase system and the chitin hydrolase system, both of which comprise a dynamic equilibrium process that is regulated by hormones and multiple signal pathways [4]. Unlike chitin research in insects, crustacean research and applications remain scarce and current efforts are still largely focused on cloning and functional analysis of single or multiple chitin-related enzyme genes. Therefore, systematic approaches to study the structural characteristics or physiological functions of chitin-associated genes in crustaceans have not been established yet.

In recent years, the rapid advancement of genomics, transcriptomics, proteomics, and other omics technologies have greatly facilitated molecular biology studies in crustacean. More than a dozen of crustacean genomes have been sequenced, including those of economically relevant decapods, such as the Pacific white shrimp *Litopenaeus* (*Penaeus*) *vannamei* [5], the black tiger shrimp *Penaeus monodon* [6], the swimming crab *Portunus trituberculatus* [7], and the marbled crayfish *Procambarus virginalis* [8], in addition to amphipods (e.g., *Parhyale hawaiensis*, and *Hyalella azteca*) [9,10], cirripeds (e.g., *Amphibalanus amphitrite*) [11], isopods (e.g., *Armadillidium vulgare*) [12], cladocerans (e.g., *Daphnia pulex* and *Daphnia magna*) [13,14] and copepods (e.g., *Eurytemora affinis* and *Tigriopus japonicus*) [15,16], among others. Moreover, a large number of RNA-Seq datasets obtained from crustaceans at different physiological states, environmental conditions, and a variety of chemical treatments can also be obtained in the public database (Table 1). The genes that encode enzymes and proteins associated with chitin metabolism can be elucidated via bioinformatics analyses. Here, we summarized several key genes and pathways related to chitin synthesis and degradation in crustaceans from a genomics standpoint to better understand the pathways associated with the chitin metabolism in crustaceans, as well as their potential applicability.

## 2. Chitin Synthesis Pathways and Genes

### 2.1. Chitin Synthesis Pathways

**Figure 1:**
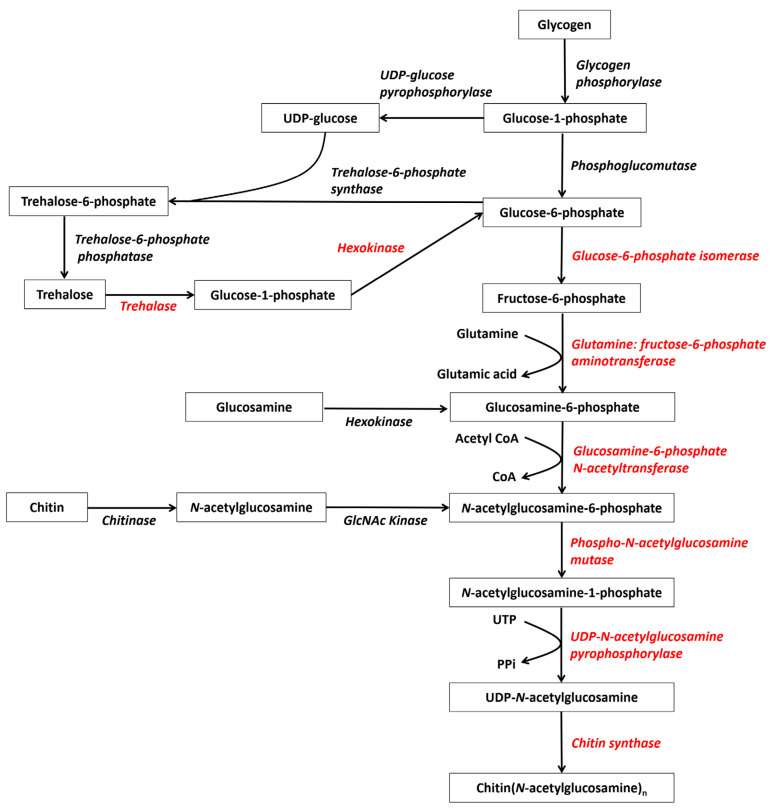
Deduced chitin synthesis pathways in crustaceans starting from glycogen, trehalose, and recycled chitin (modified from reference [19]). The red genes represent the eight enzymes participating in chitin synthesis.

The general chitin synthesis pathway is highly conserved from fungi to arthropods, and involves a series of enzymes that convert different sugars into a GlcNAc polymer [17]. In insects, trehalose is the initial raw material of chitin biosynthesis. Eight important enzymes are successively involved in this process, which begins with trehalase (TRE), followed by hexokinase (HK), glucose-6-phosphate isomerase (G6PI), glutamine: fructose-6-phosphate aminotransferase (GFAT), and glucosamine-6-phosphate *N*-acetyltransferase (GPA), 6-phosphate acetylglucosamine mutase (PAGM) and UDP-*N*-acetylglucosamine pyrophosphorylase (UDP), and finally chitin synthase (CHS) [17]. However, current studies have not comprehensively characterized the chitin biosynthesis pathways in crustaceans. Given that crustaceans and hexapods are sister groups, all of the eight aforementioned enzymes have been identified in crustacean genomes (Table 2) and their sequences are highly conserved between these two groups. Moreover, some inhibitors of insect chitin biosynthesis are also effective in crustaceans [18]. In addition, glycogen or glycogen-degraded glucose may also be the starting point for chitin synthesis in both crustaceans and hexapods, which would explain why glycogen phosphorylase and phosphoglucomutase genes have also been identified in crustaceans (Table 2). Therefore, current evidence suggests that the chitin biosynthesis pathway of crustaceans should be very similar to that of insects (Figure 1).

However, all the eight aforementioned enzymes have been completely identified only in a few crustacean species, including *L. vannamei* [5] and *D. magna* [14]. Few enzymes have been reported in most species due to genome and transcriptome data imperfections. Moreover, most chitin biosynthesis genes in crustaceans occur as single copy or two copies (Table 2), suggesting that this pathway is highly conserved. The genes that encode these eight enzymes are all important and thus attract much attention. Here, we will focus on the genes associated with three key enzymes: TRE, GFAT, and CHS.

### 2.2. Trehalase

Trehalose is a disaccharide with protein and membrane stabilizing capability that occurs naturally in plants and animals, except for vertebrates. In insects, trehalose mainly exists in the hemolymph as blood sugar, thus providing energy to fuel insect glucose metabolism. This compound is also abundant in crustacean hemolymph [20].

Trehalase hydrolyzes trehalose into two glucose molecules and is the first key enzyme in the chitin synthesis pathway. Knockdown of *SeTre1* in the beet armyworm (*Spodoptera exigua*), inhibited the synthesis of chitin in the epidermis, and interference of *SeTre2* inhibited the expression of chitinase in the midgut [21]. In *Artemia*, trehalase plays an important role in trehalose-associated metabolic processes during the formation of diapause cysts [22].

Very little information about trehalase has been reported in crustaceans [22,23]. Trehalase sequences in the GenBank database are mainly represented by the species of *D. magna*, *E. affinis*, and *Artemia*s, while many of the sequences are incomplete or present numerous similar isoforms. A small number of trehalase sequences have also been reported in isopods, barnacles, shrimp and crabs; however, the structures and functions of these genes remain largely uncharacterized.

**Figure 2:**
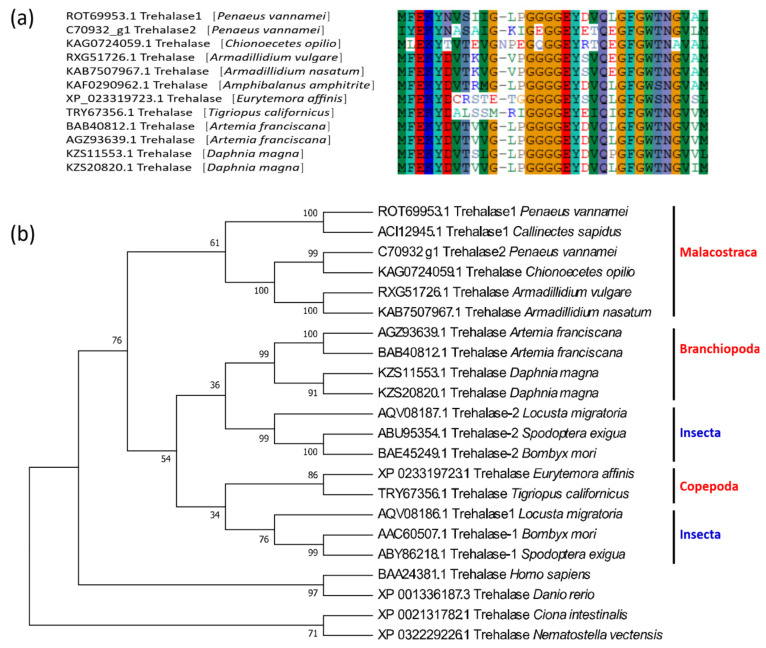
Conserved domains and phylogenetic tree of crustacean trehalase genes. (**a**) The conserved amino acids in the domains of trehalase of 12 crustaceans; (**b**) the phylogenetic tree of crustacean trehalases. The sequences are collected from the NCBI protein database, https://www.ncbi.nlm.nih.gov/protein/ (accessed on 2 January 2021).

The protein structure of crustacean trehalase is relatively conserved, and all sequences exhibit a trehalase domain. The two trehalases of *L. vannamei* share many common conserved features, including a signal peptide structure at the N-terminus, a highly conserved glutamate-rich active region (GGGGEY) [24] (Figure 2a). However, there is a transmembrane region near the C-terminus of LvTRE2, which may be a membrane-bound trehalase [23]. In contrast, LvTRE1 does not have this region (Figure S1), which indicates a functional difference between the two trehalases.

Similar to insects, two different trehalase genes, *TRE1* and *TRE2*, can be found in some crustaceans. In the trehalase phylogeny, the TRE1 and TRE2 sequences of the Malacostraca are clustered into a separate branch, insects cluster with branchiopods and copepods in two separate branches, after which these three branches cluster with chordates and cnidarians (Figure 2b).

**Figure 3:**
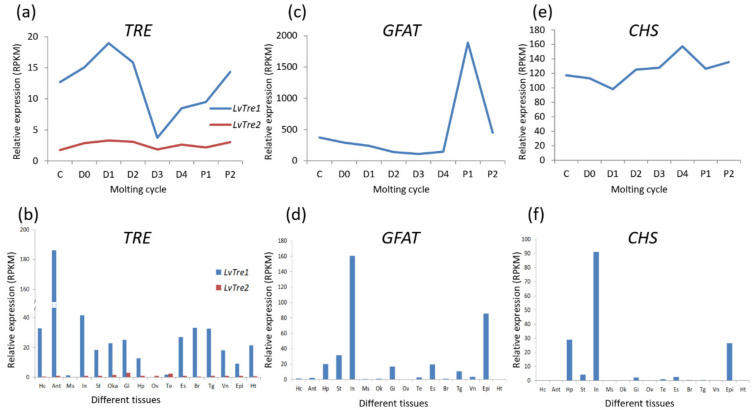
Gene-level expression of three enzymes in the chitin synthesis pathway of *L. vannamei*. (**a**) Trehalase genes (*TRE*s) expressing in molting cycle; (**b**) *TRE*s expressing in different tissues; (**c**) glutamine: fructose-6-phosphate aminotransferase gene (*GFAT*) expression in the molting cycle; (**d**) *GFAT* expression in different tissues; (**e**) chitin synthase gene (*CHS*) expression in the molting cycle; (**f**) *CHS* expression in different tissues. The bottoms of (**a**,**c**,**e**) list molting cycle of *L. vannamei*, from left to right, at the inter-molt (C) and pre-molt (D0, D1, D2, D3, D4) stages. The bottoms of (**b**), (**d**), and (**f**) list adult tissues of *L. vannamei*, from left to right, Hc: hemocyte, Ant: antennary gland, Ms: abdominal muscle, In: intestine, Ov: ovary, St: stomach, Oka: lymphoid organ, Gi: gill, Hp: hepatopancreas, Te: testis, Es: eyestalk, Br: brain, Tg: thoracic ganglion, Vn: ventral nerve, Epi: epidermis, Ht: heart. The original transcriptome data are from [25,26].

The two TRE genes identified in *L. vannamei* also exhibit differences in their expression patterns. During the molting process [25], both *LvTRE1* and *LvTRE2* have a low expression level at the D3 stage; the expression level of *LvTRE1* was high and with large fluctuations, whereas the *LvTRE2* expression was very low and had small fluctuations (Figure 3a). In adult shrimp, *LvTRE1* is highly expressed in the antennal gland, then in the intestine, muscle, and eyestalk, whereas *LvTRE2* expression is very low, mainly occurs in the gill, lymphoid organ, and testis (Figure 3b) [26]. This expression profile is different from that of insects, where *TRE1* and *TRE2* are specifically expressed in the epidermis and intestine, respectively [21]. These observations suggest that the functional differentiation of the two trehalases occurred after the differentiation of hexapods and crustaceans. However, their specific functions and regulatory mechanisms in crustaceans remain unknown.

### 2.3. Glutamine: Fructose-6-Phosphate Aminotransferase

Glutamine: fructose-6-phosphate aminotransferase (GFAT) is the first rate-limiting enzyme in the hexosamine biosynthetic pathway, which not only participates in chitin synthesis but also in many other reactions, including carbohydrate metabolism [27]. GFAT is highly conserved in bacteria, yeast, insects, and mammals. In humans, mice, fruit flies, and other organisms, two genes located in different chromosomes encode GFAT1 and GFAT2, respectively [19].

In the GenBank database, *D. magna*, *Pollicipes pollicipes*, *P. monodon*, and *L. vannamei* each possess more than 10 *GFAT* sequences; however, these sequences are isoforms of the same gene, as the two GFAT genes of *Drosophila* can be mapped to the same position in *L. vannamei*, *A. amphitrite, and D. pulex* genomes. This means that the GFAT gene of these crustaceans has numerous alternative splicing variants. Comparative analyses have demonstrated that the alternative splicing positions of *P. monodon*, and *L. vannamei* are the same and their splicing patterns are also very similar (Figure S2). In fact, the GFAT gene is so conserved between these two shrimp species that the sequences outside of the alternative splicing position have only two amino acid residues differences.

**Figure 4:**
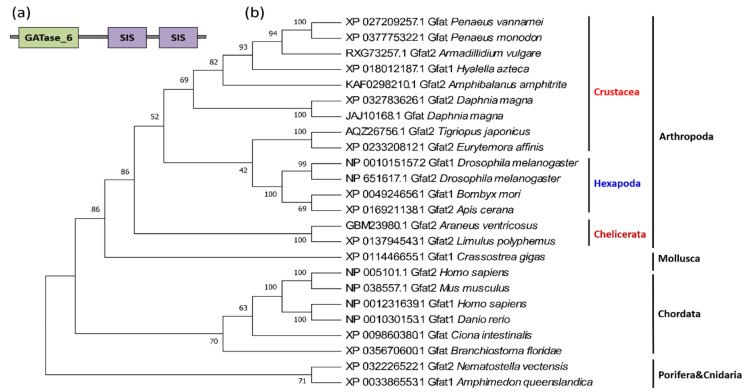
Protein domains and phylogenetic tree of crustacean GFAT genes. (**a**) Protein domain architecture of a typical GFAT gene; (**b**) the phylogenetic tree of crustacean GFAT genes. The sequences are collected from the NCBI protein database, https://www.ncbi.nlm.nih.gov/protein/ (accessed on 2 January 2021).

Phylogenetic reconstruction has demonstrated that the *GFAT*s of the malacostracans are clustered with those of cirripeds and branchiopods, then cluster with copepods and insects branch, forming a large arthropod branch with chelipods, which corresponds to another branch composed of sponges, cnidarians, and chordates (Figure 4b).

GFAT contains two structurally and functionally distinct domains, the N-terminal glutaminase domain and the C-terminal isomerase domain, the latter of which contains two similar SIS (sugar isomerase) domains, named SIS repeat I and SIS repeat II [28] (Figure 4a). The N-terminal domain and the C-terminal domain are connected by a flexible amino acid sequence to ensure that the glutaminase domain has a larger activity space during the catalytic process [28]. The glutaminase domain is responsible for the hydrolyzation of glutamine to glutamate and ammonia, as well as the transfer of the glutamine amino groups to the isomerase domain. The isomerase domain then utilizes ammonia for the conversion of fructose-6-phosphate to GlcN6P [29]. Remarkably, these domains are extremely conserved, from sponges to humans.

In insects, GFAT affects chitin metabolism and energy metabolism by regulating the hexosamine biosynthetic pathway [30]. When the GFAT gene was inhibited for 48 h in brown planthopper (*Nilaparvata lugens*), the expression of the chitin-related genes, *HK*, *GNPNA*, *UAP*, *PGMI*, *PGM2*, and *CHS* was significantly downregulated, which eventually led to a decrease in ATP content [31]. In crustaceans, GFAT is closely related to the molting process. For instance, *LvGFAT* is highly expressed at the D3 stage of the molting process of *L. vannamei* (Figure 3c). Moreover, *LvGFAT* is highly expressed in the intestine and epidermis of *L. vannamei*, but its expression is nearly absent in muscle, ovary, and heart (Figure 3d). Additionally, *LvGFAT* was strongly expressed in the hepatopancreas when the shrimp were exposed to alkaline pH conditions and cadmium stress, suggesting that this gene may play an important role in environmental stress resistance [32].

### 2.4. Chitin Synthases

Insects possess two chitin synthase (CHS) genes (*CHS1* and *CHS2*), both of which are located in the same chromosome. *CHS1* is expressed in ectoderm cells and is involved in the formation of epidermis and trachea tissue. In contrast, *CHS2* is involved in the synthesis of chitin in the intestine peritrophic matrix (PM) [33]. In crustaceans, *D. magna* has approximately 200 CHS isoforms, and more than two CHSs have been identified in cirripedes (*P. pollicipes* and *A. amphitrite*) and copepods (*L. salmonis* and *T. japonicus*). However, most decapods possess only a single copy of the CHS gene.

**Figure 5:**
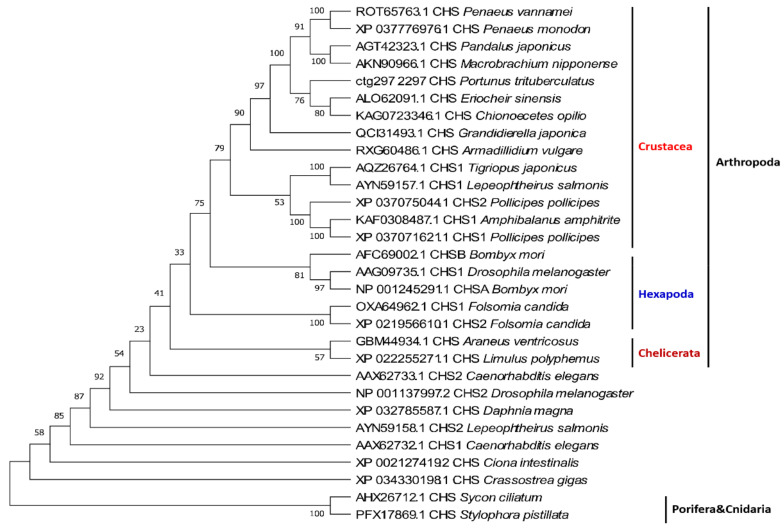
Phylogenetic tree of crustacean CHS genes. The sequences are collected from the NCBI protein database, https://www.ncbi.nlm.nih.gov/protein/ (accessed on 2 January 2021).

Phylogenetic analyses of CHS sequences have demonstrated that most crustacean CHSs cluster together, then gather with hexapods, and then cluster with chelicerates, suggesting that these CHSs are more arthropod specific. Some other arthropod CHS genes are closer to those of nematodes, mollusks, and chordates, and therefore these variants are likely more ancient (Figure 5).

**Figure 6:**
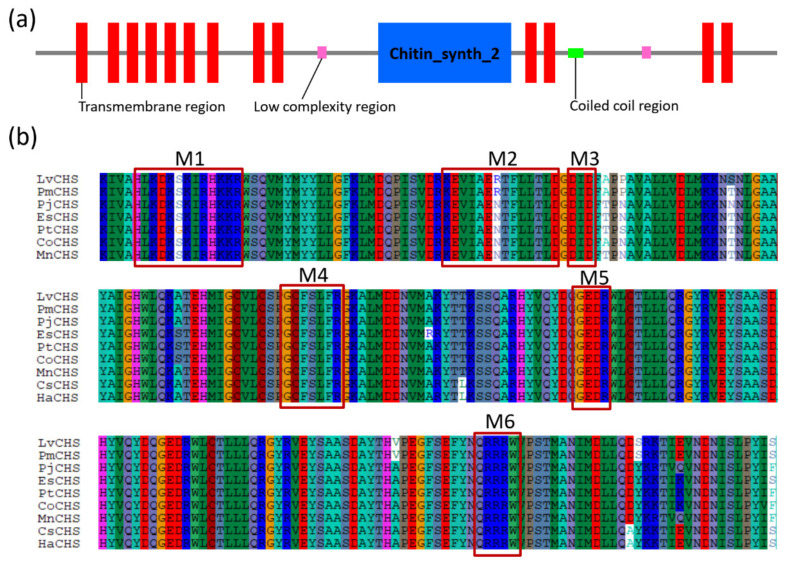
Protein domain architecture and putative catalytic domains of the CHS genes. (**a**) The protein domain architecture of CHS gene of *L. vannamei* (ROT65763.1); (**b**) the putative catalytic domains of CHS genes. LvCHS, ROT65763.1 *Litopenaeus* (*Penaeus*) *vannamei*; PmCHS, XP_037776976.1 *Penaeus monodon*; PjCHS, AGT42323.1 *Pandalus japonicus*; EsCHS, ALO62091.1 *Eriocheir sinensis*; PtCHS, ctg297 2297 *Portunus trituberculatus*; CoCHS, KAG0723346.1 *Chionoecetes opilio*; MnCHS, AKN90966.1 *Macrobrachium nipponense*; CsCHS, ALS03816.1 *Callinectes sapidus*; HaCHS, LAC20862.1 *Hyalella Azteca*. The sequences were collected from the NCBI protein database, https://www.ncbi.nlm.nih.gov/protein/ (accessed on 8 October 2020) and raw sequencing reads of *P. trituberculatus* (PRJNA555262).

The crustacean CHSs are all typical transmembrane proteins with theoretical molecular weights between 170–180 kDa and isoelectric points (Ip) between 6.1 and 6.7 (Table 2). Amino acid sequence alignment revealed that CHS has three domains (domain A, B, and C) (Figure 6a). Domain A is located at the N-terminus and consists of a number of transmembrane helixes. The number of transmembrane helices of the CHS gene varies in a species-dependent manner. These differences in the number of CHS transmembrane spirals determine the position of domain A on the cell membrane, which may be either facing the cytoplasm or outside the cell. Domain B, Chitin_synth_2 domain, is located in the middle and is the catalytic center of the enzyme. This domain contains approximately 400 highly conserved amino acids, including the two highly conserved “EDR” and “QRRRW” motifs [17] (Figure 6b). Domain C is located at the C-terminus of CHS and usually has seven transmembrane helix structures and a highly conserved motif “WGTRE”. Domain C is less conserved than Domain B but possesses two conserved amino acids (T and W) that play a catalytic role, both of which are thought to be closely related to enzyme activity [17].

CHS is a key enzyme involved in the synthesis of chitin, which is used to build the exoskeleton and peritrophic membrane. Studies on insects have demonstrated that CHS gene expression is regulated by the molting hormone, ecdysone (20-HE), and participates throughout the molting process [30]. In *L. vannamei*, *LvCHS* is widely and highly expressed in the intestine, epidermis, and hepatopancreas (Figure 3f) [34]. After molting (P1, P2 stages), *LvCHS* expression increases and then decreases during the inter-molting stage (C), after which it abruptly decreases during the pre-molting stage (D1). This is followed by a sharp upregulation from the D3 to P1 stages, indicating that the induction of *LvCHS* expression mediates the synthesis of chitin for the formation of a new exoskeleton (Figure 3e). Two CHS genes (*LsCHS1* and *LsCHS2*) were recently described in salmon louse *Lepeophtheirus salmonis*. The *LsCHS1* was conspicuously expressed in various tissues, such as the antennas, intestines, and appendages, in different development stages, whereas the highest *LsCHS2* expression was only observed in the intestine of adults [18].

Many studies have confirmed the important role of CHS in insects [32,35], however, it is unclear whether the CHS genes in crustaceans share functional roles with insects or if they are distinct. RNA interference (ds*CHS1* and ds*CHS1*+*2*) experiments in *L. salmonis* demonstrated the existence of compensation mechanisms in the chitin synthesis pathway, and CHS knockdown in these salmon lice resulted in a common phenotype, which was characterized by appendage deformities and an inability to swim [36]. Moreover, CHS inhibitors, such as diflubenzuron (DFB), hexaflumuron (HX), lufenuron (LF), and teflubenzuron (TFB) are known to interfere with chitin formation and molting in insects and completely inhibit the molting process in crustaceans (copepods), which eventually leads to death [18].

## 3. Chitin Degradation Pathways and Gene Families

### 3.1. Chitin Degradation Pathways

Chitin is mainly degraded through two biological pathways: chitin is first decomposed by chitinase (CHT) to produce oligomeric beta*-N*-acetylglucosamine (GlcNAc), after which beta-*N*-acetylglucosaminidase (NAG) further degrades the resulting oligosaccharides (poly GlcNAc) into GlcNAc monomers [30]. Another possible chitin degradation pathway involves chitin deacetylase (CDA), whereby chitin is converted into deacetylated chitins (i.e., chitosan), which are then degraded into glucosamine (GlcN) by chitosanase and glucosaminidase. These two degradation pathways often proceed simultaneously [37].

**Figure 7:**
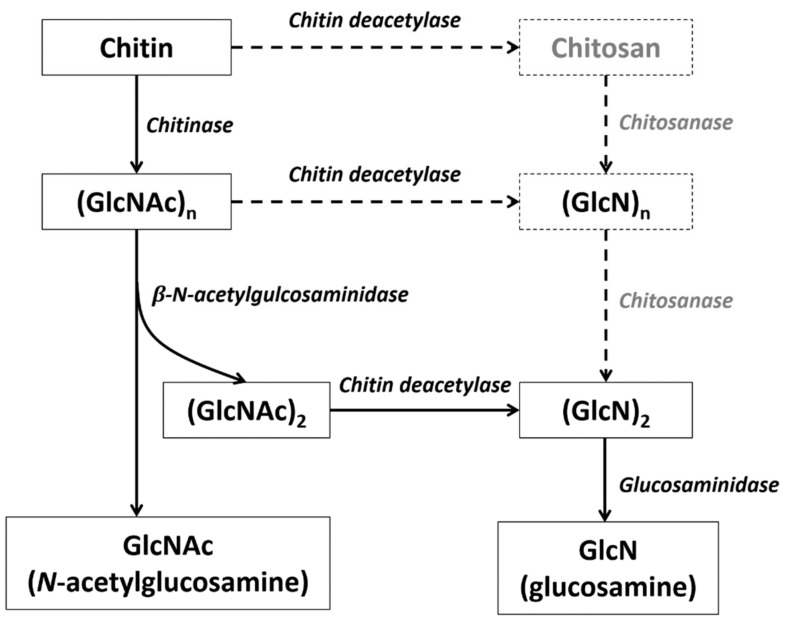
Deduced chitin degradation pathway in crustaceans. The gray enzyme and products in the dotted line box may not exist in crustaceans.

There are no systematic reports on the chitin degradation pathways in crustaceans, however, the genes of the three key enzymes involved in the first pathway (CHT, NAG, and CDA) have been identified in many species and are known to belong to multigene families. Chitosanase is another important gene for chitin degradation, however, this gene is mainly present in microorganisms (e.g., mainly fungi and bacteria) [38] and are not found in crustaceans. Glucosaminidase is another enzyme in the chitin degradation pathway and more than a dozen copies of its analogs (hexosaminidase) gene were identified in shrimp and barnacle genomes. Based on previous research in insects and the analysis of crustacean genomic data, the chitin degradation pathway of crustaceans was deduced (Figure 7).

### 3.2. Chitinase

Chitinases (CHTs) comprise a group of glycoside hydrolases that cleave β-1,4-glycosidic bonds to hydrolyze chitin into chitin-oligosaccharides (GlcNAc)_n_ and GlcNAc [39]. These enzymes have been isolated from a wide variety of sources including viruses, bacteria, fungi, nematodes, arthropods, vertebrates, and green plants, and play important roles in various physiological functions [30,38]. In crustaceans, chitinases are mainly used to regenerate or rebuild the exoskeleton, as well as for food digestion [40].

#### 3.2.1. Identification of Chitinase Genes in Crustaceans

The first crustacean chitinase was cloned from the kuruma shrimp, *Marsupenaeus* (*Penaeus*) *japonicus* [41]. Since then, many chitinase genes have been cloned in shrimp, crabs, and many other crustaceans. An increasing number of chitinase genes have been discovered with the accumulation of genome and transcriptome data. At present, more than 3000 crustacean chitinase gene sequences of crustaceans have been deposited in the Genbank database, most of which correspond to decapods with economic relevance (e.g., shrimp and crabs), as well as environmentally relevant crustaceans such as branchiopods (water fleas), copepods, amphipods, and isopods. Compared with hexapods and chelicerates, the chitinase gene has been expanded in multiple crustacean genomes (Table 3). Among them, 42 CHT genes were found in *L. vannamei* [5].

**Figure 8:**
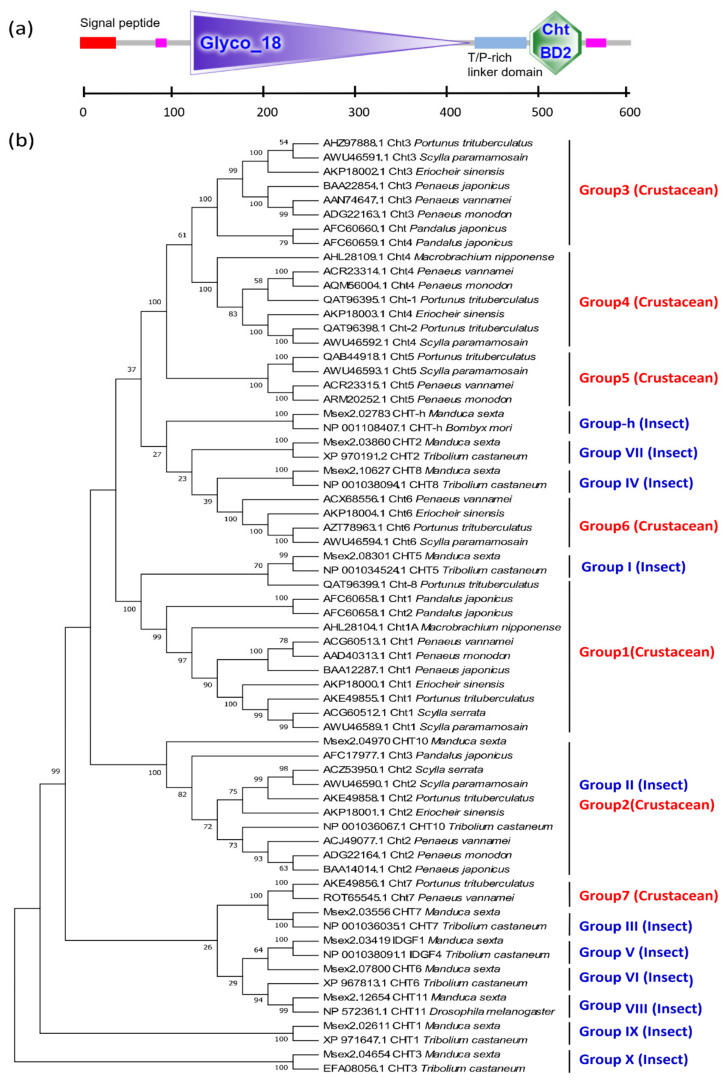
Typical GH18 domain and chitin binding domain (CBD) of CHT genes and phylogenetic tree based on chitinase amino acid sequences of crustaceans and insects. (**a**) The protein domain architecture of CHT gene of *L. vannamei*; (**b**) the phylogenetic tree based on chitinase amino acid sequences of crustaceans and insects. The sequences are collected from the NCBI protein database, https://www.ncbi.nlm.nih.gov/protein/ (accessed on 2 January 2021).

Based on amino acid sequence homology and the catalytic mechanism, chitinases can be divided into two major groups: the GH18 and GH19 families. All chitinases found in arthropods belong to the GH18 family (Figure 8a), which is a vast multigene family. In insects, the chitinase genes were originally divided into eight groups, all of which play different roles in growth and development [42]. In 2015, three chitinase gene groups (Group IX, Group X, and Group-h) were then added, thus accounting for a total of 11 groups [43].

However, research on chitinases of crustaceans remains relatively limited. Currently, the CHTs of crustaceans can be subdivided into six [40] or seven groups [44]. Group 1 and Group 3 chitinases exhibit a typical structural pattern characterized by a GH18 domain and a chitin binding domain (CBD). Group 2 chitinases have a long cDNA sequence with multiple GH18 domains and CBD domains. Group 4 chitinases have two chitin binding domains. The amino acid sequence encoded by the Group 5 chitinase gene lacks the N-terminal signal peptide sequence. Group 6 chitinases contain a part of the chitolectin catalytic domain [40]. According to phylogenetic tree analyses, Group 1 of crustaceans and Group I of insects, as well as Group 2 and Group II, are clustered together within a single group, respectively, indicating that Group 1 and Group 2 of crustaceans are closely related to the Group I and Group II of insects. Groups 3, 4, and 5 are unique to crustaceans, these cluster into a third branch; adjacent to the fourth branch, where Group 6 of crustaceans is clustered with insect Group-h, Group VII, and Group IV. Group 7 of crustaceans, which has two catalytic domains, could not be grouped into any known CHT subclasses and was therefore classified into a novel subclass [40]. In fact, the crustacean Group 7 and insect Group III have high homology and cluster together, and then cluster with Group V, Group VI, and Group VII to form a fifth branch. However, the insect Group IX and Group X of insects exhibited no crustacean homologs, suggesting that these groups are unique to insects (Figure 8b).

#### 3.2.2. Amino Acid Sequence of Crustacean Chitinase

Similar to insects, a typical crustacean chitinase has four characteristic domains: an N-terminal signal peptide (SP), a chitinase catalytic domain (CCD), a threonine/proline-rich linker domain (TPL), and a chitin binding domain (CBD) [45]. Additionally, crustacean chitinases share some common characteristics: (1) all of these chitinases exhibit the GH18 family domain; (2) the GH18 catalytic domain has four conserved motifs [44], where the second conserved Motif-II is usually thought to be the glycosylation active site; (3) the CBD domain belongs to the CBM14 family, which occurs extensively in insect peritrophic matrix proteins (PMPs) and cuticle proteins analogous to peritrophins (CPAPs) [46]; (4) the CBD structure has six conserved cysteine residues, and forms three disulfide bonds, which are believed to increase the affinity of chitinase and chitin, making these enzymes more efficient in degrading chitin; (5) a threonine/proline-rich linker domain (TPL) is commonly located between the chitin catalytic domain and the chitin binding domain of crustacean chitinase instead of the serine/threonine-rich linking domain (STL) of insects, which is most likely to be O-glycosylated, thereby increasing the stability of chitinase. This may be the main reason why chitinase can maintain its activity in protease-rich environments such as molting fluid and midgut digestive juice [45]. In summary, crustacean chitinase genes have different structural domains and different molecular weights, suggesting that they may have different functions.

#### 3.2.3. Chitinase Gene Expression Patterns

Crustacean chitinases perform three main functions. Namely, crustacean chitinases participate in the molting and growth process, the digestion of chitin-containing food, and the immune response/disease prevention. Chitinases with different functions are mainly expressed in different tissue sites. Moreover, some chitinase genes are only detected in one tissue, whereas others are found in multiple tissues.

Previous studies on the tissue expression of the chitinase gene in *L. vannamei* found that the *LvChi-5* and *LvChiD1* were expressed in the hepatopancreas, intestine, epidermis, gills, eyestalk, heart, muscle, and hemocyte; *LvChi-6* is mainly expressed in the epidermis, gills, and eyestalk; *LvChi-1*, *LvChi-3,* and *LvChi-4* are only expressed in hepatopancreas; and *LvChi-2* is mainly expressed in the eyestalk [40]. In Chinese shrimp, *Fenneropenaeus* (*Penaeus*) *chinensis*, the *FcChi-3* gene is specifically expressed in the hepatopancreas, which is both a digestive organ and the main organ for immunity in crustaceans. It is speculated that *FcChi-3* might participate in digestion and the immune response, and this conclusion has been supported by experiments such as infection with white spot syndrome virus (WSSV) infection experiments [47]. In the oriental river prawn *M. nipponense*, *MnCht4* was expressed in various tissues; however, its highest expression was observed in the intestine [48]. These findings are consistent with mud crab *Scylla paramamosain* experiments, in which the chitinase gene *SpCht6* was expressed largely in the intestine. Further studies have shown that the chitinase expressed in the hepatopancreas and intestines may be mainly related to the degradation of chitin-containing foods and nutrient absorption, whereas the chitinase gene expressed in the epidermis is mainly related to the exoskeleton’s physiological cycle or molting. Additionally, some chitinases expressed in the hepatopancreas, hemocytes and epidermis also participate in immune response mechanisms [44,49].

### 3.3. β-N-Acetylglucosaminidase

β-*N*-acetylglucosaminidase (NAG) is a type of exoglycosidase that catalyzes the hydrolysis of the oligomerization products of chitinase to generate GlcNA monomers. In insect molting fluid, the synergistic efficiency of exochitinase (NAG) and endochitinase (mainly chitinases and a few NAGs) is six times higher than that of a single NAG, and the speed of this synergistic effect depends on the proportion of the two enzymes in the molting fluid [30].

#### 3.3.1. Gene Structure of NAGs

**Figure 9:**
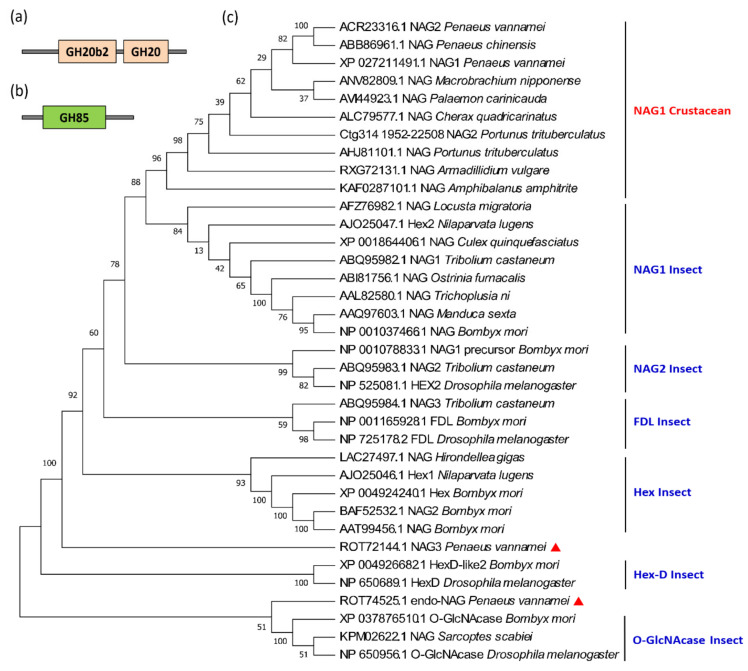
Protein domains and phylogenetic tree of the β-*N*-acetylglucosaminidase (NAG) genes in crustaceans and insects. (**a**) Protein domain of the GH18 family of NAG genes; (**b**) the protein domain of the GH85 family NAG genes; (**c**) the phylogenetic tree of NAG genes of crustaceans and insects. The red triangles show the two NAG genes of *L. vannamei* that do not belong to the NAG1 subfamily. The sequences were collected from the NCBI protein database, https://www.ncbi.nlm.nih.gov/protein/ (accessed on 2 January 2021).

In crustaceans and insects, most NAGs belong to the GH20 family. Moreover, these genes generally contain 2–4 GH20_hexosaminidase domains (Figure 9a) and have a conserved motif (HY/FGGDEV/I), in which aspartate (D) and glutamate (E) are highly conserved in the catalytic site [50]. As for the NAGs of *L. vannamei*, the carboxyl groups of acidic amino acids, imidazole groups of the histidine residue, amino groups of the lysine residue, and indole group of the tryptophan were essential for the activity of the enzyme [51]. Although *Exopalaemon carinicauda* and *M. nipponense* are closely related to penaeid shrimp based on taxonomy, their sequence homology is quite low, indicating that the NAG genes have a relatively high variation [52]. Another type of NAG is the GH85 family, including the O-GlcNAcase subfamily of insects and endo-NAGase of *L. vannamei* (Figure 9b).

NAG is a multigene family, for example, there are nine NAG genes in *Drosophila*, eleven in silkworm, and eleven in *Nilaparvata lugens*. Based on phylogenetic analyses, the insect NAG genes can be divided into six different subfamilies: NAG1, NAG2, FDL (fused lobe gene), Hex (Hexosaminidases), HexD-like, and O-G1cNAcase [53]. The NAG genes of each subfamily are clustered first, rather than being clustered according to the evolutionary relationship between species. Numerous NAG isoforms have so far been identified in the sequenced crustacean genomes (Table 3), most of them belong to the NAG1 subfamily. For example, *L. vannamei* has four NAG genes, among which *LvNAG1* and *LvNAG2* cluster with most crustaceans, and then cluster with the insect NAG1 subfamily. *LvNAG3* is between the Hex and Hex-D families, and the *Lvendo-NAGase* (ROT74525.1) clusters with insect *O-GlcNAcase*s (Figure 9c).

#### 3.3.2. Gene Expression Pattern and Function of NAGs

**Figure 10:**
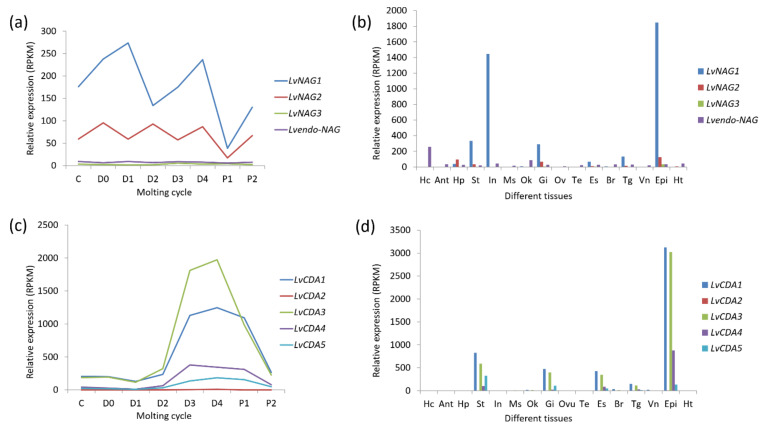
Expression patterns of NAG and chitin deacetylase (CDA) genes in different tissues and molting cycles of *L. vannamei*. (**a**) The beta-*N*-acetylglucosaminidase genes (*NAG*s) expression in the molting cycle; (**b**) the *NAG*s expression in different tissues; (**c**) the chitin deacetylase genes (*CDA*s) expression in the molting cycle; (**d**) the *CDA*s expression in different tissues. The bottoms of (**a**,**c**) list different molting stages of *L. vannamei*, from left to right, inter-molt (C) and pre-molt (D0, D1, D2, D3, D4) stages. The bottoms of (**b**,**d**) list adult tissues of *L. vannamei*, from left to right, Hc: hemocyte, Ant: antennary gland, Ms: abdominal muscle, In: intestine, Ov: ovary, St: stomach, Oka: lymphoid organ, Gi: gill, Hp: hepatopancreas, Te: testis, Es: eyestalk, Br: brain, Tg: thoracic ganglion, Vn: ventral nerve, Epi: epidermis, Ht: heart. The original transcriptome data are from [25,26].

NAG expression is known to fluctuate during the molting cycle [54]. For instance, *LvNAG1* has two peaks at the D1 and D4 stages, and its expression is low in the D2 and P1 stages of *L. vannamei*. In contrast *LvNAG2* has three peaks, of which P1 is the lowest point (Figure 10a). Similarly, in the Chinese mitten crab (*Eriocheir sinensis*) and mud crab (*Scylla serrata*) gills, high NAG activity was observed at the D0 and D3–D4 stages [55,56]. The pre-molting stage is also a period of significant change in various physiological functions and epidermal structures. As a key enzyme for chitin degradation, high NAG protein activity during this period can also ensure efficient degradation of old exoskeletons.

Previous studies have shown that crustacean epidermal NAGs are regulated by the ecdysone. In *Palaemon serratus*, *D. magna*, *U. pugilator*, and *E. sinensis*, among others, the peak value of NAG enzyme activity is positively correlated with the ecdysone content [55,57,58,59]. In the fiddler crab (*U*. *pugilator*), although the sequences of NAG derived from the epidermis and intestine are different, they all fluctuate with the molting cycle. Moreover, the ecdysone can promote the expression of the NAG gene of *U. pugilator* [60].

In the Antarctic krill *E. superba*, NAG was found in the epidermis and intestine. Particularly, the epidermal NAG is a secreted glycoprotein, whereas the intestine NAG may be mainly located in the cytoplasm [61]. In *L. vannamei, LvNAG1* is highly expressed in the epidermis, followed by the intestine. This gene has also been reported to be highly expressed in the stomach and gills, followed by the eyestalk, thoracic ganglia, and hepatopancreas. *LvNAG3* is highly expressed in the epidermis, stomach, and gills, but its expression is low in the intestine. The endo-type NAG of *L. vannamei* is highly expressed in hematocytes and the lymphatic organ (Figure 10b). In other shrimp species such as *P. serratus* and *M. japonicas*, intestinal NAG activity did not change significantly with ecdysone secretion before and after molting [57,62].

In crustaceans, some NAGs have been found to be involved in the immune response. After being challenged with the pathogens, *Vibrio parahaemolyticus* and *Aeromonas hydrophila*, the expression of *EcNAG* was upregulated significantly in *E. carinicauda* [47]. In *P. Monodon*, endo-beta-NAGase can interact with the WSSV envelope protein VP41B and thus facilitating viral infection [63]. These results indicate that NAG genes are mainly involved in the function for molting, food digestion, and immune defense mechanisms just like chitinases.

### 3.4. Chitin Deacetylase

**Figure 11:**
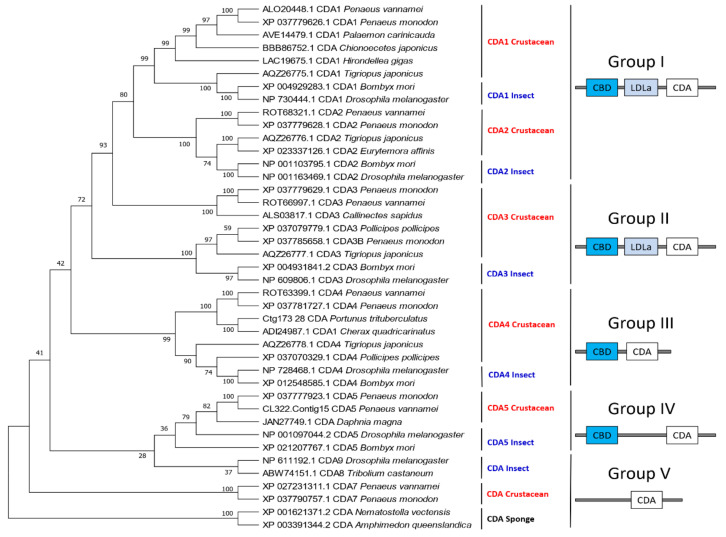
Phylogenetic tree and protein domains of different CDA groups in crustaceans and insects. The sequences were collected from the NCBI protein database, https://www.ncbi.nlm.nih.gov/protein/ (accessed on 2 January 2021).

Chitin deacetylase (CDA) belongs to the carbohydrate esterase family 4 (CE-4), which can catalyze the removal of acetyl groups from chitin [30]. There are approximately 500 crustacean CDA sequences in the GenBank database, most of which correspond to *D. magna* (423), followed by *P. monodon* (12), *T. japonicus* (10), *P. pollicipes* (8), *L. vannamei* (6). Like insects [19], the CDAs of crustaceans can also be divided into five groups based on the similarity of their amino acid sequences. The first group consists of two sub-branches, CDA1 and CDA2, both of which have three domains (CBD, LDLa and CDA). However, crustaceans and insects are clustered together according to gene sub-groups. Group II also has three domains and its main difference from Group I is the similarity and length of the genes. Both Groups III and IV have two domains (CBD and CDA), whereas Group V has a single CDA domain (Figure 11). Additionally, there are many CDA variants due to alternative splicing in some crustaceans, particularly in *D. magna*. Nonetheless, additional studies are needed to determine whether these structural differences have a functional implication.

#### 3.4.1. Structural Characteristics of CDA

Crustacean CDAs feature the CE4 superfamily domain, which contains conserved active sites of aspartate and histidine residues [64]. However, unlike insects that contain five motifs in the CE4 superfamily domain, crustaceans only have motif 1 (TFDD) and motif 2 (HSWSHP). Although all CDAs have CDA domains, they exhibit considerable differences in their amino acid sequences, the presence or absence of LDLa and ChBD domains, gene size, and their expression patterns at different developmental stages and tissues, which reflects their distinct biological functions [5,65].

#### 3.4.2. Gene Expression Pattern of CDAs

Few studies have characterized the function of CDA genes in crustaceans. However, we can infer their functions through the expression patterns of the CDAs in different molting stages and different tissues. In *P. monodon*, the *PmCDA1* was specifically highly expressed in the gills, suggesting its possible role in the immune function of shrimp against WSSV [66]. Moreover, *LvCDA*s expression is synchronous during *L. vannamei* molting cycles, they all reach a peak at D3 and D4 and then decline thereafter (Figure 10c). In adult tissues, *LvCDA1*, *LvCDA3*, and *LvCDA4* are highly expressed in the epidermis, followed by the stomach, gills, and eyestalks. *LvCDA5* is highly expressed in the stomach, followed by the epidermis and gills. The expression of *LvCDA2* is low in all tissues (Figure 10d).

## 4. Application of Chitin Metabolism Genes in Crustaceans

Chitin metabolism is a highly important and complex process. The study of these processes in crustaceans would therefore increase our understanding of the mechanisms of growth, development, reproduction, and immunity in these animals, and provide a theoretical basis for the optimization of crustacean aquaculture practices. At the same time, the chitin from crustaceans can be chemically modified to produce chitin derivatives with special properties and applications. Therefore, the study of chitin-related enzymes in crustaceans may facilitate the acquisition and utilization of these bioproducts.

### 4.1. Molting and Growth Regulation

The growth and development of crustaceans are accompanied by a periodic molting process. The completion of the molting reaction mainly depends on the dynamic balance of chitin under the combined activity of CHS and CHTs. Abnormal molting occurs when the dynamic balance between the synthesis and degradation of chitin is disrupted, or chitin content decreases [67].

A close relationship between chitinase and molting was elucidated in the 1990s after measuring chitinase activity during the molting cycle [40]. With the development of molecular biology, increasingly more genetic tools (e.g., RNAi, gene knockout) have been made available to the study of chitinase function. For instance, regulation of the molting and growth of crustaceans via manipulation of the chitinase gene has become a major breakthrough in crustacean research in recent years. In *E. sinensis*, *EsCht1* might play a role in the digestion of chitin-containing food; *EsCht2* might be essential in the degradation of chitinous cuticles during molting for growth and post-embryonic development; and *EsCht3*, *EsCht4*, and *EsCht6* were highly expressed in the reproductive system, indicating potential roles in reproductive molting [55]. These enzymes may therefore be used to promote growth and reproduction by regulating crustacean molting. Gene editing technology has also been applied in crustacean chitinase genes; for instance, the EcChi4 gene was knocked out using CRISPR/Cas9 in *E. carinicauda*, after which approximately 50% of the surviving larvae had indel mutations at the corresponding target site [68]. This technology may enable the development of novel tools for gene editing-based breeding to improve the economic traits of aquaculture animals.

The soft-shell crab has a universal appeal and a versatile flavor profile [69]. The regulation of CHS and CHT may enable the precise control of the exoskeleton remodeling process, thus making it is possible to induce synchronous molting. Therefore, additional studies on crustacean growth and molting regulation are crucial to improve the quality of aquaculture crustacean products.

### 4.2. Immune Response and Disease Control

The immune response of crustaceans such as shrimp and crabs is closely related to chitinase, which has become an indicator of virus infection as an immune-related factor. Early research found that WSSV upregulated *Pjchi-3* in the hepatopancreas of *M. japonicus*, thereby granting resistance to the virus [70]. Later studies revealed similar findings in *L. vannamei*, which were coupled with an upregulation of chitinase mRNA expression in the hepatopancreas [71]. Additionally, the detection of *LvCHT5* and *LvCHT1* in hematocytes provides insights into the involvement of *LvCHT5* and *LvCHID1* in the innate immunity of crustaceans [40]. In *LvCHT5* knock down shrimp, the expression of a large variety of immune-related genes, including transcription factors, antimicrobial peptides and other functional proteins with antibacterial and antiviral activities, was widely modified [72]. In *P. trituberculatus*, *PtCht-1*, *PtCht-2*, and *PtCht-6* may participate in the immune response against pathogens or the degradation of chitin-containing pathogens [44]. Some fungi (e.g., *Fusarium* and *Lagenidium*) are the pathogens of shrimp or crabs. Comparing the enzyme systems associated with chitin metabolism in fungi and crustaceans may lead to the identification of specific fungal target molecules that can be used to prevent the spread of these pathogens. Additionally, the cuticle is a strong pathogen barrier, and therefore the regions lacking cuticular lining such as shrimp’s antennal gland, the excretory organ, are major candidate entry portals for WSSV [73]. The above studies demonstrate that the chitin-related genes of crustaceans may participate in the immune response and disease resistance through direct or indirect regulation, thus providing important insights into pathogen control mechanisms in economically-relevant crustaceans.

Numerous studies have shown that chitin synthesis inhibitors (CSIs) can effectively interfere with arthropod chitin synthesis and molting, and are therefore widely used as pesticides in agriculture [74,75]. In salmon aquaculture, they are also used as the main method to control salmon louse (*L. salmonis*) [27]. Thus, further understanding of the roles of the various enzymes involved in chitin metabolism may provide more species-specific targets for the control of *L. salmonis* infection or other harmful crustaceans in the future [18].

### 4.3. Enzyme Engineering for the Chitin Industry

Chitosan is a copolymer of GlcNAc (~20%) and glucosamine (GlcN, ~80%) residues that results from the de-*N*-acetylation of chitin in the presence of a hot alkali [2]. Both chitin/chitosan and their modified derivatives are biodegradable, biocompatible and non-toxic, and have extensive applications in medicine, agriculture, food, cosmetics, and many other fields. Traditionally, chitin is mainly isolated from crustacean shells via demineralization with diluted acids and deproteinization with hot alkaline solutions. However, this process renders low yields and outputs high amounts of pollutants [76]. In contrast, the enzymatic degradation of chitin is environmentally friendly and renders a high product purity. Therefore, industrial chitin degradation should ideally be based on enzymatic reactions. There is a growing interest in chitinase bioengineering, as the catalytic efficiency of these enzymes is still not ideal. These challenges highlight the need for high-efficiency multifunctional chitinases. Crustaceans express a large number of chitinases with various structures. Therefore, the analysis of their domains and catalytic active sites may provide important information for enzyme activity optimization.

## 5. Conclusions

Both the chitin produced from crustaceans and the enzymes of their chitin metabolism system are very important marine biological resources. However, although crustaceans are the main sources of chitin, the study of crustacean chitin metabolism is still in its infancy. Therefore, additional research on the mechanisms of chitin biosynthesis and degradation is necessary to understand crustacean physiology and to provide a basis for the development of aquaculture and other industrial applications.

## Figures and Tables

**Table 1:** Crustacean species, the genomes and transcriptomes of which were searched for homologues of genes of the synthesis and degradation pathway.

Taxon	Species	Genome	Transcriptome	References or NCBI BioProject Accession No.
**Branchiopoda**				
Cladocera	*Daphnia pulex*	√	√	[13]
	*Daphnia magna*	√	√	[14]
Anostraca	*Artemia franciscana*		√	PRJNA449186
**Hexanauplia**				
Thoracica	*Amphibalanus amphitrite*	√	√	[11]
	*Pollicipes pollicipes*		√	PRJNA394196
Calanoida	*Eurytemora affinis*	√	√	[15]
Harpacticoida	*Tigriopus japonicus*	√	√	[16]
Harpacticoida	*Tigriopus californicus*	√	√	PRJNA237968
Siphonostomatoida	*Lepeophtheirus salmonis*	√	√	http://metazoa.ensembl.org/Lepeophtheirus_salmonis/Info/Index?db=core (accessed on 1 March 2021)
**Malacostraca**				
Amphiopoda	*Parhyale hawaiensis*	√	√	[9]
Amphiopoda	*Hyalella azteca*	√	√	[10]
Amphiopoda	*Hirondellea gigas*		√	PRJNA328366
Isopoda	*Armadillidium nasatum*		√	PRJNA249058
Isopoda	*Armadillidium vulgare*	√	√	[12]
Euphausiacea	*Euphausia superba*		√	PRJEB30084
Decapoda	*Litopenaeus (Penaeus) vannamei*	√	√	[5]
Decapoda	*Penaeus monodon*	√	√	[6]
Decapoda	*Fenneropenaeus (Penaeus) chinensis*	√	√	http://www.genedatabase.cn/fch_genome.html (accessed on 1 March 2021)
Decapoda	*Marsupenaeus (Penaeus) japonicus*;		√	PRJNA642319
Decapoda	*Macrobrachium nipponense*	√	√	PRJNA646023
Decapoda	*Procambarus virginalis*	√	√	[8]
Decapoda	*Procambarus clarkii*		√	PRJDB5062
Decapoda	*Cherax quadricarinatus*	√	√	PRJNA559771
Decapoda	*Portunus trituberculatus*	√	√	[7]
Decapoda	*Callinectes sapidus*		√	PRJNA646695
Decapoda	*Eriocheir sinensis*	√	√	PRJNA555707
Decapoda	*Scylla paramamosain*	√	√	https://figshare.com/search?q=10.6084%2Fm9.figshare.13338968 (accessed on 1 March 2021)

**Table 2:** List of genes associated with the chitin synthesis system in crustacean genomes.

Gene Name		GenBank Accession No.	Protein Length(aa)	M.W.(KDa) *	pI *
*Trehalase*	*LvTRE1*	ROT69953.1	611	70.05	5.47
	*LvTRE2*		619	71.96	7.11
	*CsTRE*	ACI12945.1	355	41.90	5.34
	*AvTRE*	RXG51726.1	605	70.15	6.13
	*AaTRE*	KAF0290962.1	583	67.39	5.77
	*AfTRE*	BAB40812.1	703	80.00	5.32
	*DmTRE*	KZS11553.1	557	63.85	4.54
*Hexokinase*	*LvHK1*	ROT76879.1	447	49.43	5.36
	*LvHK2*	ABO21409.1	484	53.28	5.63
	*PmHK*	XP_037776109.1	517	57.11	5.19
	*PtHK*	MPC12833.1	399	43.84	5.43
	*MnHK*	ASJ77422.1	448	49.72	5.29
	*DmHK*	KZS13587.1	363	39.92	5.02
	*AnHK*	KAB7498971.1	379	42.51	6.33
	*EaHK*	XP_023321614.1	558	62.35	5.45
	*PpHK*	XP_037094690.1	466	51.47	5.14
	*HaHk*	XP_018013709.1	588	65.35	4.74
*Glucose-6-phosphate isomerase*	*LvG6PI*	ROT65617.1	579	64.46	7.76
	*HgG6PI*	LAC27162.1	498	55.59	7.01
	*AvG6PI*	RXG71484.1	441	49.05	6.79
	*AaG6PI*	KAF0299334.1	556	61.31	6.84
	*TjG6PI*	AQZ26755.1	558	62.54	5.97
	*DmG6PI*	KZS17552.1	594	66.11	6.82
*Glutamine:fructose-6-phosphate aminotransferase*	*LvGFAT*	XP_027209257.1	698	78.31	6.25
	*PmGFAT*	XP_037775322.1	704	78.98	6.38
	*AvGFAT*	RXG73257.1	597	66.99	6.30
	*HaGFAT*	XP_018012187.1	586	66.35	5.84
	*AaGFAT*	KAF0298210.1	695	77.26	6.22
	*TjGFAT*	AQZ26756.1	703	78.17	6.29
	*EaGFAT*	XP_023320812.1	691	76.78	6.07
	*DmGFAT*	JAJ75170.1	588	66.50	6.65
	*DmGFAT*	XP_032783626.1	695	77.79	6.23
*Glucosamine-6-phosphate acetyltransferase*	*LvGNA*	XP_027223278.1	202	22.68	8.80
	*HaGNA*	XP_018023774.1	198	22.14	8.75
	*AaGNA*	KAF0295548.1	207	23.26	5.47
	*TjGNA*	AQZ26759.1	217	24.25	5.56
	*EaGNA*	XP_023349820.1	185	20.55	6.60
	*DmGNA*	JAM95683.1	217	24.51	6.34
*Phosphoacetylglucosamine mutase*	*LvPAGM*	ROT71834.1	536	57.45	5.69
	*AvPAGM*	RXG51983.1	476	51.57	4.98
	*AaPAGM*	KAF0304709.1	680	74.76	5.15
	*TjPAGM*	AQZ26760.1	542	59.01	5.58
	*DmPAGM*	KZS16738.1	546	59.55	5.25
*UDP-N-acetylglucosamine pyrophosphorylase*	*LvUDP*	XP_027232775.1	535	60.31	5.95
	*PmUDP*	XP_037783957.1	535	60.31	5.95
	*PtUDP*		446	49.88	6.01
	*HaUDP*	XP_018020814.1	531	59.11	6.01
	*PpUDP*	XP_037076472.1	558	62.68	5.34
	*EaUDP*	XP_023339716.1	508	56.37	5.61
	*TjUDP*	AQZ26761.1	514	57.67	5.91
	*DmUDP*	XP_032779364.1	585	66.24	7.62
*Chitin synthase*	*LvCHS*	ROT65763.1	1569	179.39	6.13
	*PmCHS*	XP_037776976.1	1565	179.48	6.40
	*PjCHS*	AGT42323.1	1525	175.01	6.12
	*EsCHS*	ALO62091.1	1574	180.64	6.74
	*PtCHS*		1479	169.67	6.45
	*MnCHS*	AKN90966.1	1566	179.57	6.09
	*GjCHS*	QCI31493.1	1521	174.07	6.14
	*AaCHS*	KAF0308487.1	1603	183.66	7.22
	*AnCHS*	KAB7494319.1	1242	142.04	6.22
	*TjCHS*	AQZ26764.1	1609	183.79	6.60
	*DmCHS*	XP_032799318.1	1533	174.88	6.03
*Glycogen phosphorylase*	*LvGP*	ROT82939.1	852	97.84	6.63
	*MjGP*	BAJ23879.1	852	98.02	6.82
	*PcGP*	AVN99053.1	849	97.47	6.63
	*AaGP*	KAF0300511.1	780	89.12	5.74
	*DmGP*	KZS14249.1	845	96.98	5.86
*Phosphoglucomutase*	*LvPGM*	XP_027216302.1	626	67.75	8.19
	*PmPGM*	XP_037793753.1	611	65.98	6.73
	*PcPGM*	AVN99056.1	561	60.84	5.53
	*HgPGM*	LAC19888.1	563	61.44	5.09
	*AaPGM*	KAF0309265.1	525	56.83	5.80
	*DmPGM*	KZS16178.1	560	61.15	5.30

* Molecular weight (M.W.) and isoelectric point (pI) were determined using the ExPASy Compute pI/Mw tool available at http://web.expasy.org/compute_pi (accessed on 6 January 2021). Lv, *Litopenaeus* (*Penaeus*) *vannamei*; Cs, *Callinectes sapidus*; Av, *Armadillidium vulgare*; Aa, *Amphibalanus amphitrite*; Af, *Artemia franciscana*; Dm, *Daphnia magna*; Pm, *Penaeus monodon*; Pt, *Portunus trituberculatus*; Mn, *Macrobrachium nipponense*; An, *Armadillidium nasatum*; Ea, *Eurytemora affinis*; Pp, *Pollicipes pollicipes*; Ha, *Hyalella azteca*; Hg, *Hirondellea gigas*; Tj, *Tigriopus japonicus*; Pj, *Pandalus japonicus*; Es, *Eriocheir sinensis*; Gj, *Grandidierella japonica*; Mj, *Marsupenaeus*(*Penaeus*) *japonicus*; Pc, *Procambarus clarkii.*

**Table 3:** Copy number of chitin degradation genes in arthropods.

Subphylum	Species	CHT Gene No.	NAG Gene No.	CDA Gene No.
Crustacea	*Litopenaeus vannamei*	42	4	5
	*Penaeus monodon*	19	2	7
	*Eriocheir sinensis*	11	6	3
	*Parhyale hawaiensis*	18	8	2
	*Amphibalanus amphitrite*	14	5	4
	*Tigriopus californicus*	8	7	5
	*Daphnia pulex*	20	6	3
Hexapoda	*Drosophila melanogaster*	13	9	9
	*Anopheles gambiae*	10	6	1
	*Apis mellifera*	10	15	3
	*Bombyx mori*	14	11	2
	*Tribolium castaneum*	24	11	12
	*Locust migratoria*	9	4	2
	*Zootermopsis nevadensis*	9	4	2
	*Acyrthosiphon pisum*	9	6	3
Myriapodia	*Strigamia maritima*	7	2	2
Chelicerata	*Ixodes scapularis*	32	1	2
	*Tetranychus urticae*	11	2	3

CHT (chitinase), NAG (beta-*N*-acetylglucosaminidase), CDA (chitin deacetylase). The original data were supplemented and modified from [5].

## Data Availability

No new data were created or analyzed in this study. Data sharing is not applicable to this article.

## Supplementary Materials

The following are available online at https://www.mdpi.com/1660-3397/19/3/153/s1, Figure S1: The protein domain architecture of trehalase genes of *L. vannamei* was predicted by SMART online software, Figure S2: The alternative splicing of GFATs in two shrimp *L. vannamei* and *P. monodon*.
