# Supplementary material for: Chitin Synthesis and Degradation in Crustaceans: A Genomic View and Application
Source: Mar Drugs. 2021 Mar 15;19(3):153. doi: 10.3390/md19030153 (PMC8002005; doi:10.3390/md19030153)
Supplement: Supplementary file 1 [file marinedrugs-19-00153-s001.pdf]

Supplementary Materials

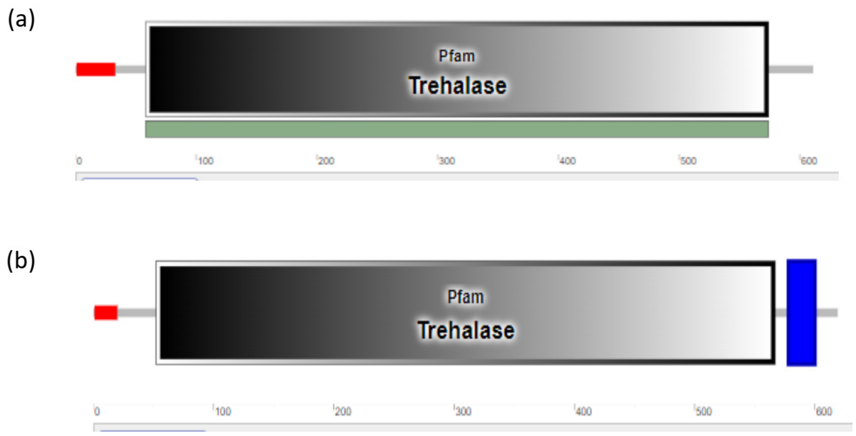

Supplement Figure 1. The protein domain architecture of trehalase genes of *L. vannamei* was predicted by SMART online program (http://smart.embl-heidelberg.de/). (a) *LvTre1*, (b) *LvTre2*.

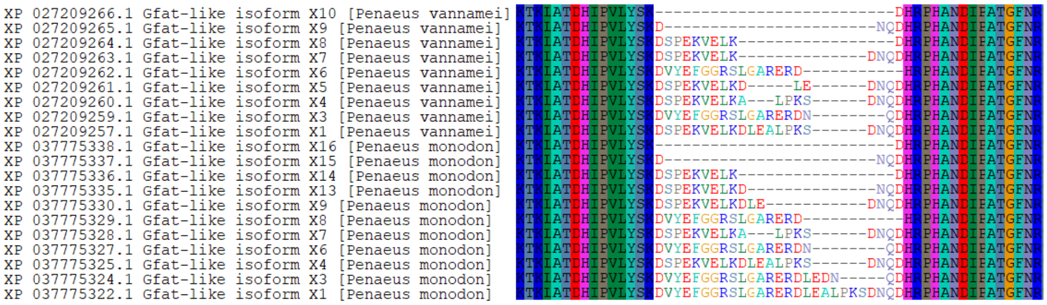

Supplement Figure 2. The alternative splicing of GFATs in two shrimp *L. vannamei* and *P. monodon*.
